# Gold Nanoparticles Downregulate IL-6 Expression/Production by Upregulating microRNA-26a-5p and Deactivating the RelA and NF-κBp50 Transcription Pathways in Activated Breast Cancer Cells

**DOI:** 10.3390/ijms25031404

**Published:** 2024-01-24

**Authors:** Aisha Farhana, Abdullah Alsrhani, Ruqaih S. Alghsham, Wassila Derafa, Yusuf Saleem Khan, Zafar Rasheed

**Affiliations:** 1 Department of Clinical Laboratory Sciences, College of Applied Medical Sciences, Jouf University, Sakaka 72388, Saudi Arabia; afalserhani@ju.edu.sa; 2 Department of Pathology, College of Medicine, Qassim University, Buraidah 51452, Saudi Arabia; r.alghasham@qu.edu.sa (R.S.A.); zafarrasheed@qu.edu.sa (Z.R.); 3 Department of Chemistry, College of Science, Jouf University, Aljouf 72388, Saudi Arabia; wderafa@ju.edu.sa; 4 Department of Anatomy, College of Medicine, Jouf University, Sakaka 72388, Saudi Arabia; dryusufkhan@gmail.com

**Keywords:** BC, GNP, gold nanoparticles, microRNA, RelA, IL-6, miR-26a-5p, NF-kB, Bay 11-7082, metal nanoparticles

## Abstract

MicroRNAs (miRNAs) are involved in the modulation of pathogenic genes by binding to their mRNA sequences’ 3′ untranslated regions (3′UTR). Interleukin-6 (IL-6) is known to promote cancer progression and treatment resistance. In this study, we aimed to explore the therapeutic effects of gold nanoparticles (GNP) against IL-6 overexpression and the modulation of miRNA-26a-5p in breast cancer (BC) cells. GNP were synthesized using the trisodium citrate method and characterized through UV-Vis spectroscopy, dynamic light scattering (DLS), and transmission electron microscopy (TEM). To predict the binding of miR-26a-5p in the IL-6 mRNA’s 3′UTR, we utilized bioinformatics algorithms. Luciferase reporter clone assays and anti-miRNA-26a-5p transfection were employed to validate the binding of miR26a-5p in the IL-6 mRNA’s 3′UTR. The activity of RelA and NF-κBp50 was assessed and confirmed using Bay 11-7082. The synthesized GNP were spherical with a mean size of 28.3 nm, exhibiting high stability, and were suitable for BC cell treatment. We found that miR-26a-5p directly regulated IL-6 overexpression in MCF-7 cells activated with PMA. Treatment of MCF-7 cells with GNP resulted in the inhibition of IL-6 overexpression and secretion through the increase of miR26a-5p. Furthermore, GNP deactivated NF-κBp65/NF-κBp50 transcription activity. The newly engineered GNP demonstrated safety and showed promise as a therapeutic approach for reducing IL-6 overexpression. The GNP suppressed IL-6 overexpression and secretion by deactivating NF-κBp65/NF-κBp50 transcription activity and upregulating miR-26a-5p expression in activated BC cells. These findings suggest that GNP have potential as a therapeutic intervention for BC by targeting IL-6 expression and associated pathways.

## 1. Introduction

BC is a complex and heterogeneous disease with various factors influencing its onset and progression [1,2]. One such factor is the cytokine interleukin-6 (IL-6), which has been implicated in BC pathogenesis. IL-6 is a pro-inflammatory cytokine that plays a crucial role in immune responses and inflammation modulation [3]. In BC, IL-6 has been shown to promote tumor growth, invasion, and metastasis through multiple mechanisms [4,5,6]. It can activate signaling pathways involved in cell proliferation, survival, and angiogenesis, contributing to tumor progression [6,7,8,9]. Additionally, IL-6 can influence the tumor microenvironment by promoting an immunosuppressive and pro-tumorigenic state [10,11,12]. Furthermore, elevated levels of IL-6 have been associated with poor prognosis and resistance to certain therapies in BC patients [9,13,14,15]. Understanding the role of IL-6 in BC onset and progression can provide valuable insights for developing targeted therapies and personalized treatment strategies for patients with this devastating disease [6,16].

MicroRNAs (miRNAs) are small non-coding RNA molecules that play a crucial role in the modulation of gene expression. They have emerged as key players in various biological processes, including cancer development and progression [17,18]. In BC, miRNAs have been extensively studied for their roles as oncogenes or tumor suppressors, contributing to the complexity of the disease [18,19]. Dysmodulation of miRNAs can lead to aberrant expression of target genes involved in cell proliferation, apoptosis, migration, and invasion, thereby promoting BC initiation and progression [20,21,22,23]. Certain miRNAs have been identified as potential diagnostic and prognostic biomarkers in BC, with their expression patterns associated with specific subtypes and clinical outcomes [24,25]. Moreover, miRNAs have shown promise as therapeutic targets, as modulation of their expression can influence tumor growth and sensitize cancer cells to treatment [26,27,28,29]. Understanding the basic biology and functional roles of miRNAs in BC provides valuable insights into the molecular mechanisms underlying the disease and holds potential for the development of novel therapeutic strategies [23,30,31,32,33].

Gold nanoparticles (GNP) have gained considerable attention in medical applications due to their unique physicochemical properties and biocompatibility. These nanoscale particles, typically ranging from 1 to 100 nanometers in size, exhibit excellent stability, high surface area-to-volume ratio, and tunable optical properties [34,35]. GNP can be easily functionalized with various molecules, making them versatile platforms for targeted drug delivery, imaging, and therapy [36,37]. In the field of cancer treatment, GNP have shown promise as vehicles for delivering chemotherapeutic drugs directly to tumor cells, enhancing drug efficacy while minimizing off-target effects [38,39]. Additionally, GNP can serve as contrast agents for various imaging techniques, including computed tomography (CT), magnetic resonance imaging (MRI), and photoacoustic imaging, providing real-time visualization of tumors and facilitating accurate diagnosis and monitoring of treatment response [40,41,42,43]. Furthermore, GNP can be utilized in photothermal therapy, where they convert absorbed light energy into heat, selectively ablating cancer cells while sparing healthy tissue [43,44]. With ongoing research and advancements, gold nanoparticles hold great potential as therapeutics and imaging agents in a wide range of medical applications, offering opportunities for personalized and precise treatments in the future [45,46]. In this study, we proposed, for the first time, that GNP have the ability to suppress the over-secretion of IL-6 through modulation mediated by miRNAs. To investigate this hypothesis, we synthesized and characterized GNP using various parameters such as zeta potential, polydispersity index, and surface plasmon resonance. We utilized bioinformatics predictions to identify a potential binding site for miR-26a-5p in the 3′ untranslated region (3′UTR) of IL-6 mRNA. To quantify the expression of miRNAs and mRNA, we employed real-time PCR assays, while sandwich ELISAs were used to measure protein secretion in the cell culture supernatant. The obtained data were further validated using luciferase reporter clone assays and transfection with anti-miRNAs. Our novel findings provided evidence that chemically synthesized GNP were not only safe but also showed therapeutic potential against the overexpression of IL-6 and the overactivation of NF-κBp65/p50 transcription activity in MCF-7 cells activated with phorbol-12-myristate-13-acetate (PMA). These findings pave the way for the development of GNP as a promising therapeutic approach to target IL-6 over-secretion.

## 2. Results

### 2.1. Properties of Chemically Engineered Gold Nanoparticles

**Figure 1:**
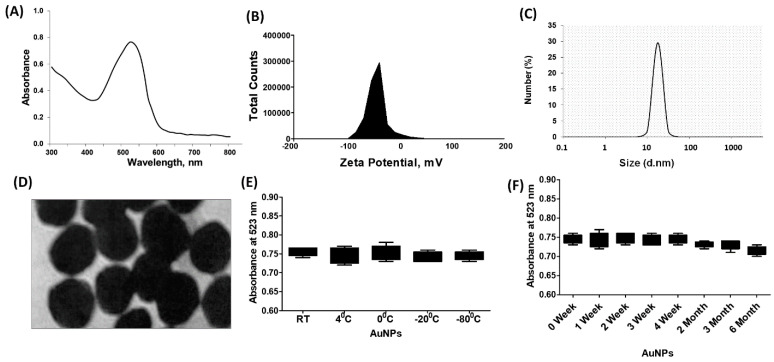
Synthesis and characterization of gold nanoparticles (GNP). (**A**) Ultraviolet-Visible (UV-Vis) spectra of GNP. (**B**) Zeta-potentialand (**C**) particle size distribution of GNP determined by dynamic light scattering. (**D**) Transmission electron microscopy (TEM) image of GNP. (**E**) Determination of stability of GNP against varying temperatures. (**F**) Determination of stability of GNP against storage time.

The chemically engineered gold nanoparticles were characterized using various analytical techniques. UV-Vis spectroscopy was performed in the wavelength range of 300–800 nm, and the surface plasmon resonance (SPR) peak was observed at 523 nm (Figure 1A). To assess the stability of the nanoparticles, aliquots of the gold nanoparticles were stored at different temperatures for 6 months. After this period, the SPR peak reading at 523 nm remained consistent for all aliquots, indicating that the nanoparticles were stable at room temperature (−80 °C to 4 °C) (Figure 1B). In addition, the stability of the nanoparticles was tested over a 6-month storage period at 4 °C, and the readings at 523 nm were similar for all aliquots (Figure 1C). The structure of the gold nanoparticles was examined using transmission electron microscopy (TEM), revealing a spherical shape (Figure 1D). Dynamic light scattering (DLS) analysis determined the average size of the nanoparticles to be 28.3 nm (Figure 1E), with a zeta potential of −32.2 mV (Figure 1F). The polydispersity index (PPI) of the nanoparticles was 0.435, suggesting a relatively narrow size distribution. Furthermore, the stability of the newly synthesized gold nanoparticles was tested in a cell culture supernatant (DMEM-F12) containing various components. The nanoparticles were incubated in this medium for 24 h at 37 °C, and TEM images confirmed their stability and overall structure, which matched the images shown in Figure 1D.

### 2.2. Cytotoxicity Assays of GNP against Cancer Cells Viability

**Figure 2:**
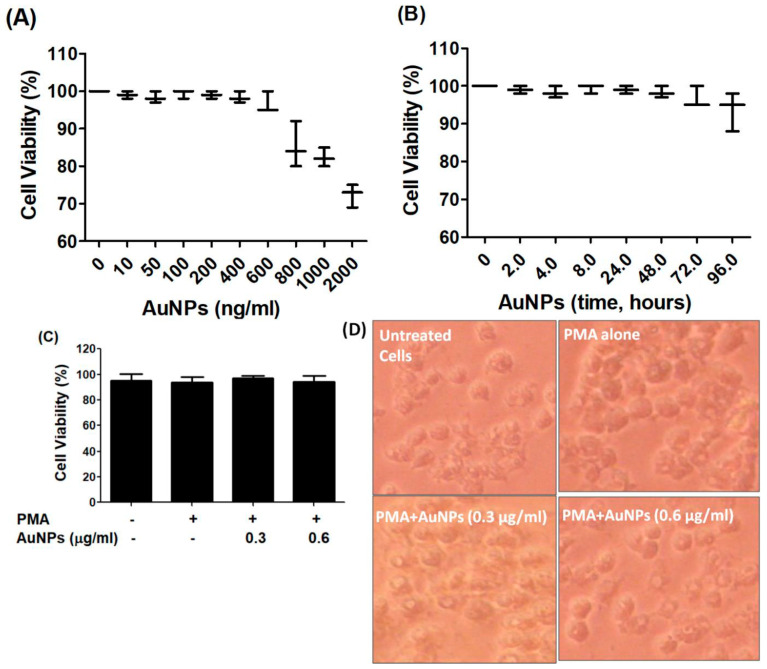
GNP on the viability of MCF-7. (**A**) Percent viability of MCF-7 cells (3 × 10^6^ cells/mL) against GNP (0–2000 ng/mL) treatment for 48 h. (**B**) Percent viability of MCF-7 cells (3 × 10^6^ cells/mL) against GNP (600 ng/mL) for 0–96 h treatment time. (**C**) Percent viability of PMA-activated MCF-7 cells (3 × 10^6^ cells/mL) treated or untreated with GNP for 24 h. (**D**) Phenotypic evaluation PMA-activated MCF-7 cells treated or untreated with GNP for 24 h. The data shown are mean ± SD of five independent experiments.

**Figure 3:**
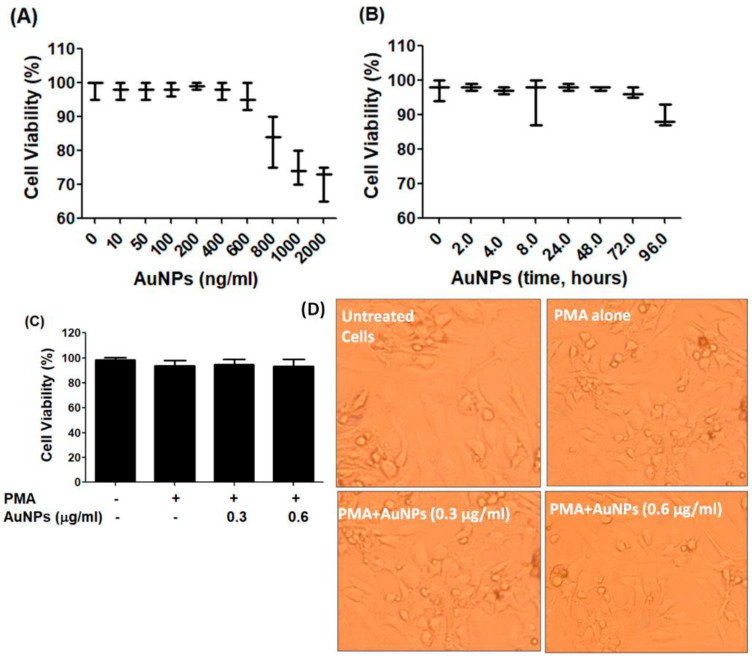
GNP on the viability of MDA-MB-231. (**A**)Percent viability of MDA-MB-231 cells (3 × 10^6^ cells/mL) against GNP (0–2000 ng/mL) treatment for 48 h. (**B**) Percent viability of MDA-MB-231 cells (3 × 10^6^ cells/mL) against GNP (600 ng/mL) for 0–96 h treatment time. (**C**) Percent viability of PMA-activated MDA-MB-231 cells (3 × 10^6^ cells/mL) treated or untreated with GNP for 24 h. (**D**) Phenotypic evaluation PMA-activated MDA-MB-231 cells treated or untreated with GNP for 24 h. The data shown are mean ± SD of five independent experiments.

The BC cells MCF-7, which were serum starved, were treated with newly engineered GNP (ranging from 0 to 2 µg/mL) for 24 h. The Luminescent-based Viability Assay kit by Promega Corporation was used to quantify the cell viability. The results indicated that the cancer cells remained stable up to a treatment of 0.6 µg/mL GNP, after which their viability percentage began to decline (refer to Figure 2A). To validate the stability of the cancer cells after treatment with 0.6 µg/mL GNP, they were exposed to this concentration for periods ranging from 2 to 72 h. Viability percentage was quantified using the same Luminescent Cell Viability Assay kit, and the results displayed in Figure 2B clearly showed that 0.6 µg/mL GNP did not exhibit toxicity towards the cancer cells over the 72 h treatment period. Based on these findings, the range of 0.3–0.6 µg/mL GNP was standardized and used for treatment in MCF-7 cells for all subsequent experiments. To assess the cytotoxicity of GNP in PMA-activated MCF-7 cells, the cells were treated with GNP (0.3–0.6 µg/mL) for 2 h before being activated with PMA (0.1 µM) for 24 h. Treatment with PMA alone or in combination with GNP did not exhibit any toxicity (*p* > 0.05; refer to Figure 2C). Additionally, bright field microscopic images of MCF-7 cells treated with PMA and GNP were analyzed to assess any phenotypic effects. As illustrated in Figure 2D, the MCF-7 cells did not show any morphological or phenotypic alterations. To verify the cytotoxicity of PMA and GNP in other BC cell types, the MDA-MB-231 cell line was used. This cell line is considered one of the best BC cell lines besides MCF-7. Using the same experimental conditions as with MCF-7 cells (Figure 2), all cytotoxicity assays were repeated using the Luminescent-based Viability Assay kit by Promega in MDA-MB-231 cells. The results shown in Figure 3A–D clearly demonstrate that GNP or PMA did not exhibit cytotoxic effects on MDA-MB-231 cells.

### 2.3. Human Cytokine Antibody Array and Western Blotting

**Figure 4:**
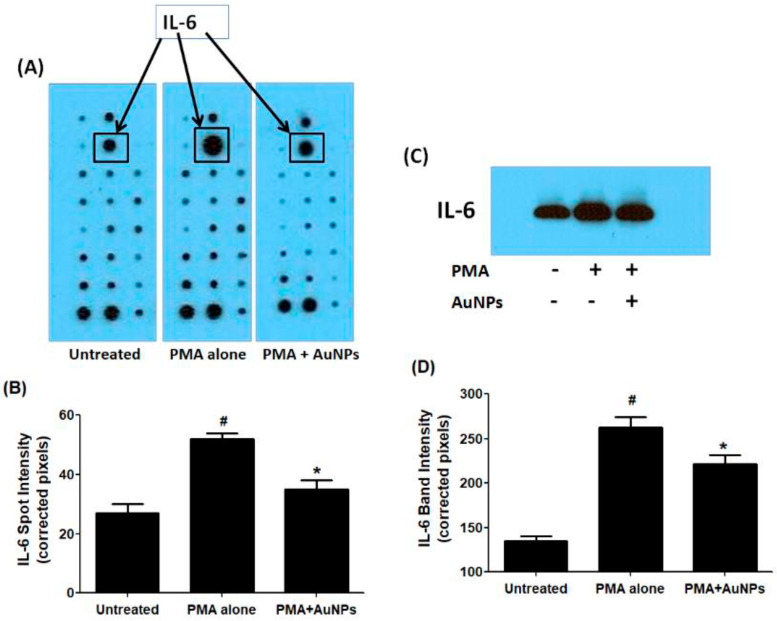
IL-6 secretion by PMA-activated human BC cell line MCF-7 cells treated or untreated with GNP. (**A**)The levels of IL-6 in the pooled culture supernatant (*n* = 3) of PMA-activated MCF-7 cells treated or untreated with GNP (0.6 µg/mL) for 24 h were quantified by human cytokine antibody array. (**B**) IL-6 spot image was digitally captured by the Un-Scan-It software and the band intensities were expressed in corrected pixels with background correction. (**C**) The levels of IL-6 in the pooled culture supernatant (*n* = 3) of PMA-activated MCF-7 cells treated or untreated with GNP (0.6 µg/mL) for 24 h were quantified by Western immunoblotting. (**D**) IL-6 band image intensity was digitally captured by the Un-Scan-It software and the band intensities were expressed in corrected pixels with background correction. # *p* < 0.05 versus untreated; # *p* < 0.05 versus *.

To determine the secretion of cytokines, a commercial human cytokine antibody-based array (RayBio Human Cytokine Array C5, RayBiotech, Norcross, GA, USA) was utilized. Human BC cells were pretreated with GNP (0.6 µg/mL) for 2 h and then activated with PMA for 24 h. The culture supernatant from three independent experiments was collected, pooled, and used for the array experiment following the manufacturer’s recommendations. The analysis focused specifically on the constitutive expression of major cytokines out of the 80 proteins including cytokines, chemokines, and growth factors present on the array. Un-Scan-It software Version 5.1 by Silk Scientific Corporation (Orem, UT, USA) was used to analyze the IL-6 spots. The data demonstrated that all major cytokines were produced by MCF-7 cells, with the secretion level of IL-6 significantly enhanced after 24 h of PMA treatment (*p* < 0.05) compared to the secretion levels of other cytokines (refer to Figure 4A,B). Remarkably, the data also indicated that the altered secretion of IL-6 protein induced by PMA was significantly suppressed by GNP (*p* < 0.05). The strips from all three sets of experimental arrays containing IL-6 protein are shown in Figure 4A, while the quantitative measurement of IL-6 in terms of spot intensities is depicted in Figure 4B.

To validate these findings regarding IL-6 protein secretion in the culture supernatant of treated and untreated MCF-7 cells, western immunoblotting was performed. The data presented in Figure 4C,D confirmed that treatment of MCF-7 cells with GNP (0.6 µg/mL) prior to PMA treatment significantly reduced PMA-activated IL-6 secretion (*p* < 0.05). These results were also corroborated by the quantitative measurement of IL-6 band intensity using Un-Scan-It software (Figure 4D).

### 2.4. Computer-Based Prediction of miRNAs in 3′UTR of Human IL-6 mRNA and Their Experimental Validation

**Figure 5:**
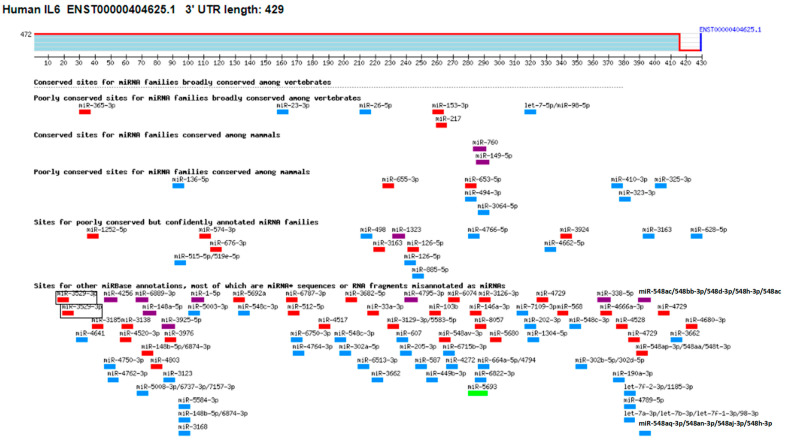
Target scanning of seed matched sequence of miRNAs in the 3′UTR of human IL-6. Human IL-6 mRNA 3′UTR (ENST00000404625.1) predicts conserved site for <100 miRNAs. The figure was generated using an online bioinformatic software Targetscan using the link https://www.targetscan.org/cgi-bin/vert_72/view_gene.cgi?rs=ENST00000404625.1&taxid=9606&members=&subset=1&showcnc=1&shownc=1&shownc_nc=1&showncf1=1&showncf2=1#miR-365-3p (accessed on 22 October 2023).

**Figure 6:**
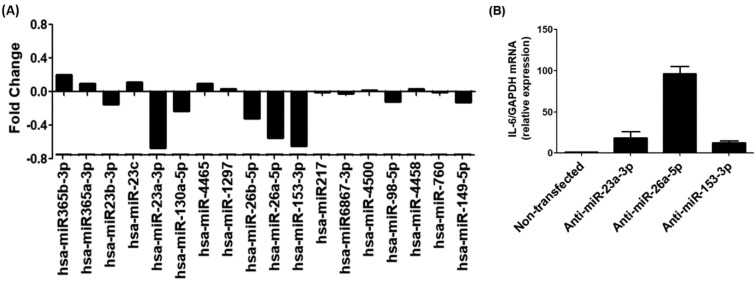
Experimental validation of bioinformatics prediction of highly scored miRNAs in the IL-6 3′UTR. (**A**) Taqman assays for miR365b-3p, miR365a-3p, miR-23b-3p, miR-23c, miR-23a-3p, miR-130a-5p, miR-4465, miR-1297, miR-26b-5p, miR-26a-5p, miR-153-3p, miR-217, miR3867-3p, miR-4500, miR-98-5p, miR-4458, miR-760 and miR-149-5p in PMA-activated MCF-7 cells. The obtained expression profile of specific miRNA in PMA-activated cancer cells were compared with untreated cells and the data presented in fold change. (**B**) Expression of IL-6 mRNA in MCF-7 cells transfected with anti-miR-23a-3p, anti-miR-26a-5p or anti-153-3p.

The TargetScan bioinformatics algorithm was used to identify seed-matched sequences of all potential miRNAs in the 3′UTR of human IL-6 mRNA (ENST00000404625.1). The 3′UTR sequence of human IL-6 mRNA contained 429 nucleotide bases (Figure 5). Multiple miRNA sequences displayed complementarity with the IL-6 3′UTR sequence. Based on specific bioinformatics predicted parameters such as duplex binding site type, context score, score percentile, weight context score, conserved branch length, probability of conserved targeting PCT, and predicted relative KD, 18 miRNA complementary sequences with IL-6 3′UTR were chosen for experimental validation. The eight selected miRNA sequences were miR365b-3p, miR365a-3p, miR-23b-3p, miR-23c, miR-23a-3p, miR-130a-5p, miR-4465, miR-1297, miR-26b-5p, miR-26a-5p, miR-153-3p, miR-217, miR3867-3p, miR-4500, miR-98-5p, miR-4458, miR-760, and miR-149-5p. To validate these predictions made through computer-based analysis, the expressions of specific miRNAs were quantified individually using specific TaqMan assays in PMA-activated MCF-7 cells, and the results were compared with untreated MCF-7 cells (Figure 6A). Only three miRNAs, namely miR-23b-3p, miR-26a-5p, and miR-153-3p, exhibited more than a 0.5-fold change in down-modulation compared to untreated MCF-7 cells. Consequently, these three miRNAs were selected for further investigation. To determine the optimal miRNA target for IL-6 modulation, MCF-7 cells were transfected with anti-miR-23b-3p, anti-miR-26a-5p, or anti-miR-153-3p. The results demonstrated that transfection of MCF-7 cells with anti-miR-26a-5p led to a significant up-modulation of IL-6 mRNA (*p* < 0.05) compared to non-transfected MCF-7 cells (Figure 6B). In contrast, transfection of MCF-7 cells with anti-miR-23b-3p or anti-miR-153-3p resulted in negligible up-modulation of IL-6 mRNA. Hence, the miRNA miR-26a-5p was ultimately selected for further studies.

### 2.5. MicroRNA-26a-5p Regulates IL-6 Expression in Cancer Cells

**Figure 7:**
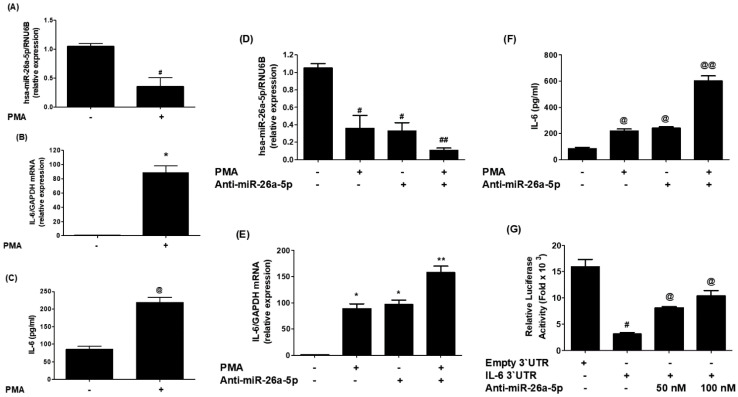
MicroRNA miR-26a-5p negatively correlated with IL-6. (**A**)Expression of miR-26a-5p in PMA-activated BC cells. (**B**) PMA-activated IL-6 mRNA in cancer cells. (**C**) PMA-activated IL-6 secretion in the cell culture supernatant. (**D**) PMA-activated miR-26a-5p in anti-miR-26a-5p transfected cancer cells. (**E**) PMA-activated IL-6 mRNA in anti-miR-26a-5p transfected cells. (**F**) PMA-activated IL-6 in the culture supernatant of anti-miR-26a-5p transfected cells. (**G**) Luciferase activity in cancer cells transfected with the reporter vector containing entire 3′UTR of IL-6 mRNA (IL-6 3′UTR) and anti-miR-26a-5p. Bars with different symbols are statistically significant, *p* < 0.05.

To further validate the predicted binding of miR-26a-5p in the IL-6 3′UTR and the findings from Section 2.3, experiments were conducted as shown in Figure 7. In Figure 7A, it can be observed that the expression of miR-26a-5p was significantly down-regulated (*p* < 0.05) in MCF-7 cells activated with PMA. Conversely, the IL-6 mRNA was significantly up-regulated upon PMA stimulation (*p* < 0.001; Figure 7B). Subsequently, the cell culture media of PMA-treated and non-treated MCF-7 cells were analyzed with IL-6 specific sandwich ELISAs to test whether the effect on IL-6 mRNA also led to IL-6 protein secretion. The results, presented in Figure 7C, demonstrate a significant increase in IL-6 secretion in response to PMA stimulation (*p* < 0.01), indicating elevated levels of IL-6 at both the mRNA and protein levels. To confirm the inverse correlation between miR-26a-5p and IL-6 expression, MCF-7 cells were transfected with anti-miR-26a-5p and subsequently activated with PMA. As depicted in Figure 7D, transfection with anti-miR-26a-5p significantly down-regulated miR-26a-5p expression (*p* < 0.01), similar to the effect seen when non-transfected cells were activated with PMA alone. Notably, a significant decrease in miR-26a-5p expression was observed when anti-miR-26a-5p-transfected MCF-7 cells were treated with PMA (*p* < 0.05). Interestingly, transfection with anti-miR-26a-5p significantly up-regulated IL-6 mRNA (*p* < 0.01), mirroring the effect observed when non-transfected cells were activated with PMA alone. Similarly, a marked up-modulation in IL-6 mRNA was found when anti-miR-26a-5p-transfected MCF-7 cells were treated with PMA (*p* < 0.05; Figure 7E). Additionally, the cell culture media of transfected and non-transfected MCF-7 cells were analyzed, revealing a significant increase in IL-6 protein secretion when cancer cells were transfected with anti-miR-26a-5p (*p* < 0.01). This effect was similar to the increased IL-6 protein secretion seen when non-transfected cells were activated with PMA alone. Intriguingly, a remarkable over-secretion of IL-6 protein was observed when anti-miR-26a-5p-transfected MCF-7 cells were treated with PMA (*p* < 0.05; Figure 7F). These results clearly indicate an inverse correlation between miR-26a-5p expression and IL-6 expression and secretion. To investigate whether this inverse correlation is mediated through IL-6 3′UTR, co-transfection of MCF-7 cells with the IL-6 3′UTR reporter clone and anti-miR-26a-5p was performed. The relative luciferase activity was compared to the cancer cells transfected with the IL-6 3′UTR reporter clone alone. As shown in Figure 7G, co-transfection of cancer cells with IL-6 3′UTR reporter clone + anti-miR-26a-5p significantly increased relative luciferase activity in a dose-dependent manner of anti-miR-26a-5p, compared to the cancer cells transfected with the IL-6 3′UTR reporter clone alone. These findings confirm that microRNA-26a-5p directly regulates IL-6 expression/secretion by binding to the IL-6 3′UTR in MCF-7 BC cells.

### 2.6. GNP Inhibit IL-6 mRNA and Protein through miR-26a-5p in Cancer Cells

**Figure 8:**
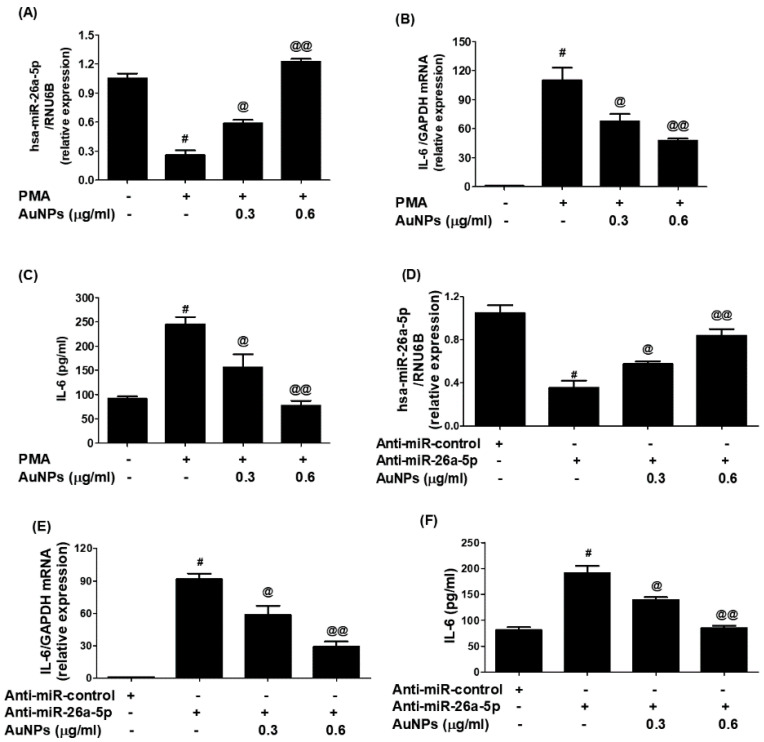
Modulation of expression of miR-26a-5p and IL-6 by GNP. (**A**) GNP increase miR-26a-5p in PMA-treated cancer cells. (**B**) GNP suppressed expression of IL-6 mRNA in PMA-activated cancer cells. (**C**) GNP suppressed IL-6 in the culture supernatant of PMA-activated cancer cells. (**D**) GNP increased miR-26a-5p in cancer cells transected with anti-miR-26a-5p. (**E**) GNP suppressed IL-6 mRNA in cancer cells transected with anti-miR-26a-5p. (**F**) GNP suppressed IL-6 secretion by the cancer cells transected with anti-miR-26a-5p. Bars with different symbols are statistically significant, *p* < 0.05.

In an effort to study the therapeutic potential of newly engineered GNP in controlling pathogenic genes through miRNA modulation, researchers conducted experiments on BC cells (MCF-7). The cells were treated with varying concentrations of GNP (0.3–0.6 µg/mL) before being activated with PMA. The results (Figure 8A) showed that PMA stimulation downregulated the expression of miR-26a-5p, while pretreatment with GNP prior to PMA stimulation significantly upregulated its expression in a dose-dependent manner (*p* < 0.05). Furthermore, the same cancer cells activated with PMA showed a significant increase of IL-6 mRNA, whereas pretreatment with GNP prior to PMA stimulation dose-dependently downregulated IL-6 mRNA (*p* < 0.05; Figure 8B). Similarly, the researchers examined the effect of GNP on IL-6 protein secretion in the same MCF-7 cancer cells treated with GNP and PMA. The pattern of IL-6 protein secretion mirrored that of IL-6 mRNA levels. PMA stimulation significantly increased IL-6 protein secretion, but pretreatment with GNP significantly inhibited IL-6 protein levels in the cell culture supernatant (*p* < 0.05; Figure 8C). To further validate the therapeutic potential of GNP, the researchers transfected MCF-7 cancer cells with anti-miR-26a-5p or anti-miR-control and then treated them with GNP in varying doses. The results (Figure 8D) demonstrated that transfection with anti-miR-26a-5p downregulated miR-26a-5p expression, whereas treatment with GNP significantly upregulated its expression in a dose-dependent manner (*p* < 0.05). Conversely, transfection with anti-miR-26a-5p upregulated IL-6 mRNA, but treatment with GNP dose-dependently inhibited IL-6 mRNA levels (*p* < 0.05; Figure 8E). Likewise, the effect of GNP on IL-6 protein secretion in the same transfected MCF-7 cells showed that treatment with anti-miR-26a-5p significantly enhanced IL-6 protein secretion, while treatment with GNP inhibited IL-6 protein levels in the culture supernatant (*p* < 0.05; Figure 8F).

**Figure 9:**
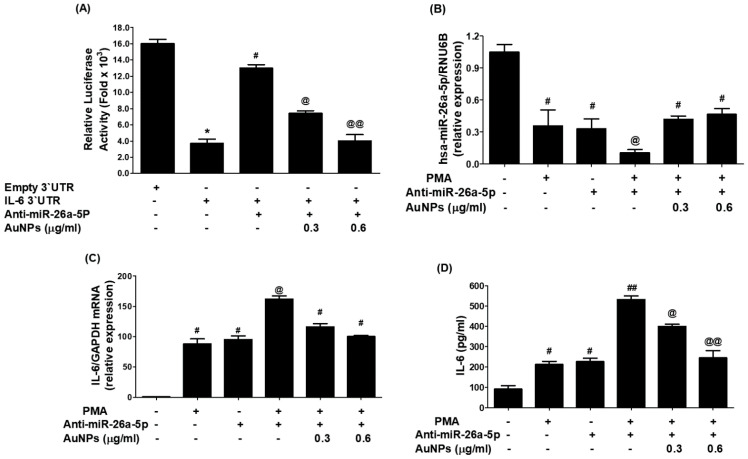
GNP suppressed IL-6 by upregulating miR-26a-5p.(**A**) GNP on the luciferase activity in cancer cells simultaneously transfected with IL-6 3′UTR reporter vector + anti-miR-26a-5p. (**B**) GNP increased miR-26a-5p expression in anti-miR-26a-5p-transfected breast cells activated with PMA. (**C**) GNP suppressed IL-6 mRNA in anti-miR-26a-5p-transfected cancer cells activated with PMA. (**D**) GNP suppressed IL-6 in the culture supernatant of anti-miR-26a-5p-transfected cancer cells activated with PMA. Bars with different symbols are statistically significant, *p* < 0.05.

To evaluate whether GNP upregulated miR-26a-5p and inhibited IL-6 mRNA/protein through IL-6 3′UTR, the researchers conducted co-transfection experiments with MCF-7 cells using IL-6 3′UTR reporter clone + anti-miR-26a-5p. The data in Figure 9A showed that co-transfection significantly increased relative luciferase activity compared to cells transfected with the IL-6 3′UTR reporter clone alone (*p* < 0.001). However, treatment of these cells with GNP decreased relative luciferase activity in a dose-dependent manner, confirming that GNP inhibit IL-6 expression or upregulate miR-26a-5p through IL-6 3′UTR. To further support these results and confirm the therapeutic potential of the newly engineered GNP, anti-miR-26a-5p-transfected MCF-7 cells were pretreated with GNP and then activated with PMA. The expression levels of miR-26a-5p and IL-6 mRNA/protein were determined. Results in Figure 9B showed that transfection with anti-miR-26a-5p alone downregulated miR-26a-5p expression (*p* < 0.01), similar to the decline observed in non-transfected cells treated with PMA alone. However, a notable decline in miR-26a-5p expression was observed when anti-miR-26a-5p-transfected cells were treated with PMA (*p* < 0.05). This decline was reversed when the cells were treated with GNP before PMA treatment. Interestingly, levels of IL-6 mRNA were significantly higher (*p* < 0.05) in lysates of the same anti-miR-26a-5p-transfected cells treated with GNP prior to PMA treatment (Figure 9C). Similarly, the protective effects of GNP were observed at the IL-6 protein level in the culture supernatants of anti-miR-26a-5p-transfected MCF-7 cells treated with GNP prior to PMA treatment (Figure 9D). These results validated that GNP suppress IL-6 mRNA/protein through miR-26a-5p expression.

### 2.7. GNP Attenuate PMA-Activated RelA and NF-κBp50

**Figure 10:**
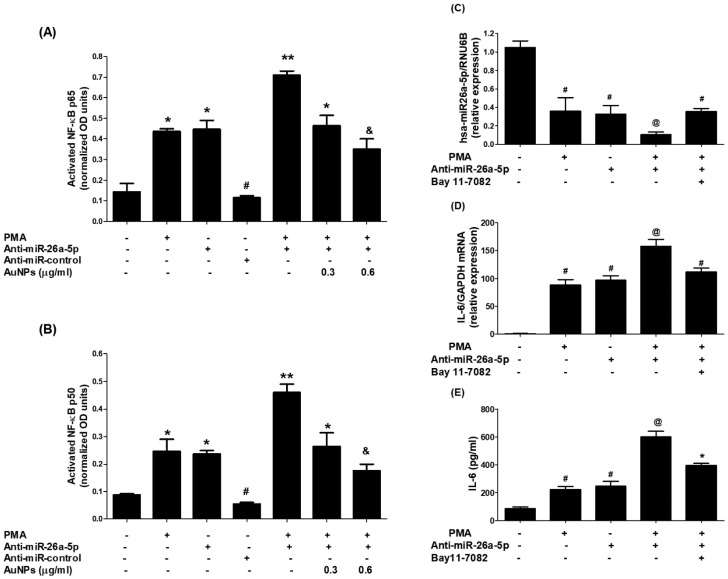
GNP suppressed PMA-increased RelA and NF-κBp50 activation through miR-26a-5p. (**A**) GNP suppressed PMA-increased RelA activation. (**B**) GNP suppressed PMA-activated NF-κBp50 in BC cells. (**C**) Bay 11-7082 increased miR-26a-5p in anti-miR-26a-5p-transfected cancer cells treated with PMA. (**D**) Bay 11-7082 suppressed IL-6 mRNA in anti-miR-26a-5p-transfected cancer cells treated with PMA. (**E**) Bay 11-7082 suppressed IL-6 induction in the culture supernatant of anti-miR-26a-5p-transfected cancer cells activated with PMA. Bars with different symbols are statistically significant, *p* < 0.05.

The nuclear extracts of MCF-7 cells were analyzed to understand the cellular pathways involved in the inhibition of IL-6 induced by GNP through miR-26a-5p increase. In the experiment, the levels of activated RelA (a component of the master transcription factor) and NF-κBp50 were examined in the nuclear extracts of MCF-7 cells transfected with anti-miR-26a-5p and treated with GNP and PMA. The results indicated that stimulation of MCF-7 cells with PMA significantly increased the level of activated RelA. An increased level of activated RelA was observed in the nuclear extracts of MCF-7 cells transfected with anti-miR-26a-5p. Interestingly, there was a marked increase in the level of activated RelA in the nuclear extract of anti-miR-26a-5p-transfected MCF-7 cells activated with PMA. Importantly, treatment of these cells with GNP prior to PMA significantly inhibited the level of activated RelA in a dose-dependent manner (Figure 10A). In another set of experiments, the level of activated NF-κBp50 was examined. Stimulation of MCF-7 cells with PMA significantly increased the level of activated NF-κBp50. Similar to RelA, an increased level of activated NF-κBp50 was observed in the nuclear extracts of MCF-7 cells transfected with anti-miR-26a-5p. Moreover, a marked increase in the level of activated NF-κBp50 was found in the nuclear extract of anti-miR-26a-5p-transfected MCF-7 cells activated with PMA. However, when these cells were treated with GNP prior to PMA, the level of activated NF-κBp50 was significantly inhibited in a dose-dependent manner (Figure 10B).

To re-confirm the involvement of NF-κB activity in the modulation of IL-6 expression and miR-26a-5p, an NF-κB inhibitor called Bay 11-7082 was used. Ani-miR-26a-5p-transfected MCF-7 cells were pretreated with Bay 11-7082 and then activated with PMA. It was observed that the expression of miR-26a-5p declined when these cells were treated with PMA. However, this decline was reversed when the cells were treated with Bay 11-7082 prior to PMA (Figure 10C). Additionally, the levels of IL-6 mRNA were significantly higher in the lysate of the same set of cells treated with Bay 11-7082 prior to PMA (Figure 10D). Similar protective effects of Bay 11-7082 were observed at IL-6 protein levels in the culture supernatants of these cells when they were treated with GNP before PMA (Figure 10E). In short, these results demonstrate that GNP can attenuate PMA-induced expression and secretion of IL-6 by increasing the level of miR-26a-5p and inhibiting RelA and NF-κBp50 in human BC cells.

## 3. Discussion

This study demonstrates for the first time that trisodium citrate-generated GNP can reduce the expression of interleukin-6 (IL-6) mRNA and protein induced by PMA in human BC cells. BC is a widespread and significant form of cancer that affects millions of people globally. While it is more common among women, men can also develop BC, albeit at lower rates [47]. The global prevalence of BC makes it a major public health concern, as reported by the World Health Organization (WHO), which estimated 2.3 million new cases diagnosed in 2020 alone [48]. The incidence of BC varies across regions, with higher rates observed in developed countries. Various risk factors contribute to the development of BC, including advancing age, family history of the disease, certain genetic mutations like BRCA1 and BRCA2, hormonal factors, lifestyle choices, and environmental exposures [49]. Early detection through regular breast self-examinations, clinical examinations, and mammography screenings is crucial for improving survival rates. Thanks to increased awareness, improved access to healthcare, and advancements in treatment strategies, better outcomes have been achieved for BC patients [50,51]. However, it is essential to continue efforts to gain a deeper understanding of the disease, develop effective prevention strategies, and provide optimal care for those affected by BC [52,53]. In this study, we used a commercial human cytokine antibody-based array to investigate the secretion of cytokines in human BC cells (MCF-7). The cells were pre-treated with GNP (0.6 µg/mL) for 2 h and then activated with PMA for 24 h. The culture supernatant from three independent experiments was collected, pooled, and subsequently used for the array experiment according to the manufacturer’s guidelines. Our analysis focused on the constitutive expression of major cytokines among the 80 proteins, including cytokines, chemokines, and growth factors present on the array. The data revealed that MCF-7 cells produced all major cytokines; however, the secretion of IL-6 exhibited a significant increase upon 24 h PMA treatment in comparison to the secretion levels of other cytokines. To identify microRNAs with the potential to regulate IL-6 expression, we performed computer-based prediction of seed-matched sequences in the 3′UTR of human IL-6 mRNA. Utilizing the TargetScan bioinformatics algorithm, we identified a 429-nucleotide base sequence in the 3′UTR of human IL-6 mRNA, which was used for the prediction. Several miRNA sequences were found to be complementary to the IL-6 3′UTR sequence, based on bioinformatics predictions, taking into account various parameters such as duplex binding site type, context score, score percentile, weight context score, conserved branch length, the probability of conserved targeting PCT, and predicted relative KD. Among the predicted miRNA sequences, eighteen were selected for experimental validation. For experimental validation, we quantified the expression of specific miRNAs using miRNA Taqman assays in PMA-activated MCF-7 cells and compared the results with untreated MCF-7 cells. The data revealed that only three miRNAs, namely miR-23b-3p, miR-26a-5p, and miR-153-3p, exhibited a down-modulation of more than 0.5-fold compared to untreated MCF-7 cells. Based on these differential expressions, we selected miR-23b-3p, miR-26a-5p, and miR-153-3p for further investigation. To determine the most effective miRNA for regulating IL-6 expression, we transfected MCF-7 cells with anti-miR-23b-3p, anti-miR-26a-5p, or anti-miR-153-3p. Our findings demonstrated that transfection with anti-miR-26a-5p significantly up-regulated IL-6 mRNA compared to non-transfected MCF-7 cells. In contrast, transfection with anti-miR-23b-3p or anti-miR-153-3p resulted in negligible up-modulation of IL-6 mRNA. Therefore, we selected miR-26a-5p for further investigation as the miRNA target for regulating pathogenic expression of IL-6. IL-6, a cytokine with various functions in cancer development and progression, is produced by different cell types including immune cells, fibroblasts, and tumor cells. Its effects on cancer cells and the tumor microenvironment are diverse [8]. It can activate signaling pathways like STAT3 and MAPK to promote cancer cell survival, proliferation, and invasion. IL-6 also stimulates angiogenesis, which is crucial for tumor growth and metastasis [7,8]. Additionally, it has immunomodulatory properties by inhibiting T cell function and favoring the expansion of regulatory T cells [11]. IL-6 has been associated with the epithelial-to-mesenchymal transition (EMT) process in cancer cells as well [54,55]. The dysmodulation of IL-6 signaling is observed in various cancers, such as breast, lung, liver, and prostate cancer. Considering its multiple effects, IL-6 has emerged as a potential therapeutic target in cancer treatment [56]. Inhibition of IL-6 signaling pathways or neutralization of IL-6 itself has shown promise in preclinical and clinical studies, emphasizing the importance of targeting IL-6 for effective cancer therapy. MicroRNAs also play a significant role in BC. They regulate gene expression post-transcriptionally and their dysmodulation can affect critical cellular processes like proliferation, apoptosis, migration, and invasion. Certain miRNAs have been identified as oncogenes or tumor suppressors, contributing to the complexity of the disease [26,27]. They can promote tumor initiation or regulate oncogene expression and cell cycle arrest [19,57,58]. Moreover, miRNAs are involved in the modulation of EMT, a crucial process in cancer metastasis [18,19]. Altered miRNA expression profiles have been associated with BC subtypes, clinical outcomes, and therapeutic responses [27,34,35]. Understanding the role of miRNAs in BC can provide insights into the underlying molecular mechanisms and aid in the development of diagnostic tools and targeted therapies for improved patient outcomes. Recently, the role of microRNA-26a-5p was discovered as a negative regulator of various pathogenic genes in different human disorders. It has been shown to suppress cancer proliferation marker genes in several cancer types, including BC [34,58,59]. The therapeutic potential of microRNA-26a-5p has been highlighted as it inhibits the expression of multiple pathogenic genes in various human cancer cells [58,59]. However, its role against IL-6 in any cell type, including cancer cells, has not been explored. In this study, for the first time, it was reported that microRNA-26a-5p directly regulates IL-6 in human BC cells activated with PMA. The study demonstrated that microRNA-26a-5p regulates IL-6 expression through complementary pairing in the 3′UTR of IL-6 mRNA sequences. This was validated through bioinformatic analysis and experimental studies using BC cells. The analysis predicted a complementary sequence between microRNA-26a-5p and IL-6 3′UTR, supporting the formation of a duplex. In MCF-7 cells activated with PMA, a down-modulation of microRNA-26a-5p expression and an up-modulation of IL-6 mRNA and protein secretion were observed, showing an inverse correlation between microRNA-26a-5p and IL-6. Transfection with anti-microRNA-26a-5p confirmed this inverse correlation. Co-transfection with IL-6 3′UTR reporter clone and anti-microRNA-26a-5p enhanced relative luciferase activity compared to transfection with the IL-6 3′UTR reporter clone alone. These findings suggest that microRNA-26a-5p directly regulates IL-6 through IL-6 3′UTR. Overall, this study provides valuable insights into the direct modulation of IL-6 by microRNA-26a-5p in human BC cells, highlighting its potential as a therapeutic target.

Nanotechnology involves manipulating matter at the nanoscale which has ushered in a paradigm shift in various fields, including medicine. In the realm of cancer biology, one particularly promising application is the use of gold nanoparticles. These nanoparticles possess unique physical and chemical properties that make them perfect candidates for targeted drug delivery, imaging, and therapy in cancer treatment. By attaching specific ligands, GNP can selectively target cancer cells, improving drug delivery while minimizing side effects [60,61]. In addition, their large surface area allows for the attachment of therapeutic agents like chemotherapy drugs or gene therapies, enhancing the effectiveness of treatment [62,63]. GNP can also serve as imaging agents, aiding in precise tumor diagnosis and monitoring of progression [64]. Furthermore, their photothermal properties can be harnessed for photothermal therapy, where GNP absorb light energy and convert it into heat, selectively killing cancer cells [64]. The therapeutic role of gold nanoparticles in cancer biology holds immense promise, paving the way for more effective and personalized treatment strategies in the future. This study aimed to explore the potential of GNP in suppressing IL-6 over-secretion in BC cells and to investigate whether this inhibition is mediated by the modulation of miRNAs. To this end, GNP were chemically synthesized, characterized, and their cytotoxicity was assessed on MCF-7 cells. Results from UV-Vis spectroscopy, TEM, DLS, and cytotoxicity assays revealed that the newly synthesized GNP were spherical with an average size of 28.3 nm, highly stable, and non-toxic to MCF-7 cells as well as MDA-MB-231 cells.

As mentioned earlier, miR-26a-5p co-regulates the overexpression of IL-6 in BC cells. Therefore, targeting miR-26a-5p could serve as a novel therapeutic approach to monitor IL-6 overexpression in BC. In this study, we targeted miR-26a-5p using chemically generated GNP to assess the levels of IL-6 in activated BC cells. We treated MCF-7 cells with different concentrations of GNP prior to PMA stimulation. Results demonstrated that GNP treatment significantly enhanced miR-26a-5p expression and dose-dependently inhibited IL-6 at both the mRNA and protein levels. Further validation of these therapeutic potentials was conducted by transfecting MCF-7 cells with anti-miR-26a-5p. The findings showed that transfection of MCF-7 cells with anti-miR-26a-5p down-regulated miR-26a-5p expression. However, treatment with GNP significantly upregulated miR-26a-5p expression and dose-dependently inhibited IL-6 mRNA and protein levels. To investigate whether the increase of miR-26a-5p and inhibition of IL-6 mRNA/protein by GNP involved IL-6 3′UTR, co-transfection experiments were performed using MCF-7 cells with an IL-6 3′UTR reporter clone and anti-miR-26a-5p. The results revealed that co-transfection significantly increased relative luciferase activity, indicating regulatory effects of anti-miR-26a-5p. However, treatment with GNP significantly decreased relative luciferase activity in a dose-dependent manner, confirming that GNP inhibit IL-6 expression and upregulate miR-26a-5p via IL-6 3′UTR. Collectively, these findings confirm that GNP inhibit IL-6 mRNA/protein expression by upregulating miR-26a-5p.

**Figure 11:**
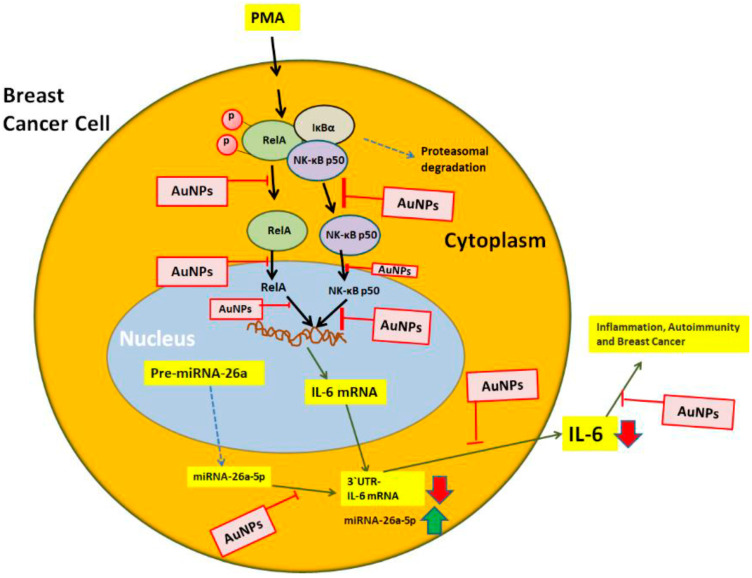
Therapeutic illustration of GNP potential against IL-6 induced inflammation, autoimmunity and BC. PMA activates RelA and NF-κB50 mediated signaling to IL-6 via miR-26a-5p. The figure systematically illustrates all the potential targets through which GNP can inhibit IL-6. The NF-κB complex, consisting of RelA and NF-κB50 subunits, is depicted, while the pathways indicated by broken arrows were not investigated. The abbreviations used are GNP for gold nanoparticles, PMA for phorbol-12-myristate-13-acetate, NF-κB for nuclear transcription factor-kappa B, and IL-6 for interleukin-6.

The master transcription factor NF-κB is a complex consisting of five proteins, including p65 (RelA) and p50. This critical transcription factor has been found in almost all human cell types, where it plays a crucial role in cell survival by regulating DNA transcription and cytokine secretion [65,66,67]. However, under pathological conditions, the over-activation of NF-κB can lead to the over-secretion of inflammatory mediators. These mediators have been directly linked to the onset of various human disorders, including cancer. Normally, NF-κB proteins form a complex in the cytoplasm of cells. However, upon receiving a signal from external stimuli, such as PMA, the NF-κB complex undergoes proteasomal degradation and the dissociated NF-κB proteins, like RelA and p50, become active. They then translocate to the nucleus, where they regulate DNA transcription and mRNA synthesis [68,69,70]. While studies have shown that the NF-κB signaling pathway is involved in the overexpression of IL-6 in cancer cells [71,72], the role of NF-κB in IL-6 overexpression/secretion through miRNA modulation has not been thoroughly investigated. This study aimed to target the NF-κB pathways (RelA and p50) to determine whether their activation could be controlled by GNP. Specifically, under inflammatory conditions, we analyzed the cellular pathways involved in GNP-induced inhibition of IL-6 via increase of miR-26a-5p. To better understand these signaling events, the components of the NF-κB pathways (RelA and NF-κBp50) were analyzed in the nuclear extracts of anti-miR-26a-5p-transfected MCF-7 cells treated with GNP and PMA. The overall events studied in this research are presented in Figure 11. The findings showed that stimulation of MCF-7 cells with PMA or transfection with anti-miR-26a-5p led to a significant increase in the levels of activated RelA and NF-κBp50 subunits. Interestingly, a marked increase in the levels of activated RelA and NF-κBp50 was observed in the nuclear extracts of anti-miR-26a-5p-transfected MCF-7 cells activated with PMA. However, treatment with GNP significantly inhibited the levels of activated RelA and NF-κBp50 in a dose-dependent manner. To confirm the involvement of RelA and NF-κBp50 activities in the modulation of IL-6 expression and miR-26a-5p, we used the well-known NF-κB inhibitor Bay 11-7082 [73]. Anti-miR-26a-5p-transfected MCF-7 cells were pretreated with Bay 11-7082, followed by stimulation with PMA, and the levels of miR-26a-5p and IL-6 mRNA/protein were determined. Our data showed that Bay 11-7082 treatment resulted in an increase in miR-26a-5p expression and a decrease in the levels of IL-6 mRNA and secreted IL-6 protein in anti-miR-26a-5p-transfected MCF-7 cells treated with PMA. These findings provide clear evidence that GNP attenuate IL-6 expression and secretion by increasing the level of miR-26a-5p and deactivating RelA and NF-κBp50 in human BC cells. In view of the findings of this study described in a mechanistic manner in Figure 11, it is also important for us to interpret the mechanism and show the problems in extrapolating its results to actual BC. It is documented that PMA and NF-κB are both involved in the development and progression of various cancers, including BC [70,74,75,76]. PMA is a potent tumor-promoting agent that can activate the NF-KB pathway, leading to the expression of genes that promote cell survival, proliferation, invasion, and angiogenesis, whereas NF-κB itself is a transcription factor that plays a crucial role in the modulation of genes involved in immune responses, cell survival, and inflammation. In BC, aberrant activation of NF-κB has been reported in both cancer cells and the surrounding tumor microenvironment [74,75,76]. This activation can contribute to tumor growth, metastasis, and resistance to chemotherapy. PMA can stimulate NF-KB activity, potentially exacerbating these effects. Therefore, understanding the relationship between PMA, NF-κB, and BC progression provide insights into novel therapeutic strategies, which actually we investigated in this study. However, it’s important to note that treating BC involves a multifaceted approach, and targeting specific molecular pathways such as NF-κB is just one aspect of this complex disease. Although PMA treatment in MCF-7 cells has provided valuable insights into BC biology, it is important to acknowledge the limitations and challenges in extrapolating these findings to actual BC scenarios. These limitations include the synthetic nature of PMA, heterogeneity within BC subtypes, and optimizing treatment concentration and duration. In view of these, we used optimal concentrations of PMA in this study which was 0.1M for 24 h, which is non-toxic to MCF-7 as well as MDA-MB-231 (Figure 2C and Figure 3C). These findings are very important as the concentration and duration of PMA treatment can significantly impact the observed outcomes. High concentrations of PMA may induce non-physiological responses and cause cytotoxic effects. It is also important to point out that the caution must be exercised when extrapolating the findings from PMA treatment of MCF-7 cells to actual BC scenarios. One critical concern is the applicability of PMA as an accurate representation of endogenous activators present in the natural physiological microenvironment. PMA is a synthetic compound that may not fully recapitulate the diverse and complex signaling mechanisms observed in actual BC cells. Furthermore, BC is a heterogeneous disease with distinct molecular subtypes and characteristics. Although MCF-7 cells have been valuable models in BC research, they may not fully encompass the entirety of BC variations. Therefore, extrapolating findings from PMA treatment in MCF-7 cells to diverse BC subtypes may not fully capture the complexities observed in actual patient samples.

## 4. Material and Methods

### 4.1. Preparation and Characterization of Gold Nanoparticles

The synthesis of GNP was carried out using the trisodium citrate method, following a previously described protocol [34,57,77]. To initiate the synthesis, a solution of 26.2 mM trisodium citrate dihydrate (Na_3_C_6_H_5_O_7_·_2_H_2_O; molecular weight: 294.10) was prepared in distilled water. Simultaneously, chloroauric acid (HAuCl_4_, 2 mM) was dissolved in 10 mL of boiled distilled water, and 4 mL of the prepared trisodium citrate dihydrate solution was added to it. The reaction mixture, composed of the combined trisodium citrate dihydrate and chloroauric acid solutions, was boiled for 1.5 h. After boiling, the mixture was cooled to room temperature and then subjected to centrifugation. The supernatant obtained from the centrifugation was collected and used as the GNP solution.

The synthesized GNP underwent several characterizations to assess their properties. ultraviolet-visible (UV-Vis) spectroscopy was performed to determine the surface plasmon resonance (SPR) peak. Moreover, the structure and shape of the GNP were examined using transmission electron microscopy (TEM), following a previously reported protocol [78]. The size of the newly synthesized GNP was determined through the quantification of the polydispersity index (PDI) and zeta potential, using dynamic light scattering (DLS) techniques as outlined in previous studies [57,77]. Overall, these characterization methods provided valuable insights into the properties and morphology of the synthesized GNP.

### 4.2. Human BC Cells MCF-7 and MDA-MB-231

The MCF-7 and MDA-MB-231 human BC cell lines were purchased from the ATCC, and their culture followed the manufacturer’s guidelines. In the case of MCF-7 cells, they were cultured in complete DMEM-F12 media with the addition of 10% heat-inactivated fetal bovine serum (FBS), 100 U/mL of MEM nonessential amino acids, 100 U/mL of penicillin, and 100 U/mL of streptomycin. The culture media also included insulin at a concentration of 10 μg/mL. These cells were grown in a 5% CO_2_ incubator at 37 °C. On the other hand, the MDA-MB-231 cells were cultured under the same conditions as the MCF-7 cells, with one exception. Insulin was not used in the culture media for MDA-MB-231 cells. Everything else, including the high glucose DMEM medium, 10% heat-inactivated FBS, and penicillin (100 U/mL) and streptomycin (100 μg/mL) concentrations, remained the same. These cells were also grown at 37 °C in a 5% CO_2_ and 95% air environment.

### 4.3. Stimulation of MCF-7 and MDA-MB-231 Cells with PMA and GNP Treatment

The cancer cells, which were approximately 70–80% confluent, were first serum starved for 12 h. Afterwards, they were subjected to treatment with PMA at a concentration of 0.1 µM, along with different concentrations of GNP (ranging from 0.0 to 2000 ng/mL) in various experimental setups. To assess the viability of the treated cancer cells, the luminescent-based cell viability assay kit (Promega, Madison, WI, USA) was used. Once the viability of the cells was confirmed, the serum-starved MCF-7 cells were pre-treated with GNP at concentrations between 0.3 and 0.6 µg/mL for a duration of 2 h. Following this pre-treatment, the cells were activated with 0.1 µM PMA (Sigma-Aldrich, Saint Louis, MO, USA) for 24 h. On the other hand, untreated cancer cells were used as a negative control in accordance with previous studies [79].

### 4.4. Human Cytokine Antibody Array

We used a commercial human cytokine antibody-based array (RayBio Human Cytokine Array C5, RayBiotech, Norcross, GA, USA) to measure the secretion of cytokines. They conducted their experiments on human BC cells, where the cells were first treated with GNP (at a concentration of 0.6 µg/mL) for a duration of 2 h. Following this, the cells were activated with PMA for 24 h. To perform the cytokine array experiment, the culture supernatant from three independent experiments was collected, pooled together, and used as per the manufacturer’s recommendations. The obtained IL-6 spots were then analyzed using the Un-Scan-It software provided by Silk Scientific Corporation.

### 4.5. Bioinformatics Approach

The bioinformatics tool Target Scan (http://www.targetscan.org/) was utilized on 11 October 2023 to predict the seed match sequences of miRNAs in the 3′UTR of IL-6 mRNA. This approach relies on validated computer-based algorithms, known for accurately predicting the complementary pairing of miRNAs with pathogenic mRNA sequences within the 3′UTR [80].

### 4.6. Transfection of Cancer Cells with miRNA Inhibitors and Treatment with PMA or GNP

The BC cells were transfected with anti-miRNAs (microRNA inhibitors, Ambion/Qiagen, Austin, TX, USA) using HiPerfect Transfection Reagent (Qiagen, Germantown, MD, USA). After 72 h post-transfection, the transfected cancer cells were pretreated with GNP (0.3–0.6 µg/mL) for 2 h. Subsequently, they were activated with PMA (0.1 µM) for 24 h. The cell culture supernatants were saved, and cell lysate or RNA samples were prepared for further analysis, as described previously [81].

### 4.7. Luciferase Reporter Assays

The binding of miR-26a-5p in the 3′UTR of IL-6 mRNA was validated using luciferase reporter assays as described previously [34].

### 4.8. Real Time PCR and TaqMan Assays

The levels of mRNA and miRNAs in the cancer cells, both treated and non-treated, were quantified using a technique called quantitative PCR (qPCR). Specific TaqMan assays from Applied Biosystems (Waltham, MA, USA) were used along with the StepOne real-time PCR system from Life Technologies (Carlsbad, CA, USA). To begin, total RNA from the cancer cells was isolated using the mirVana miRNA isolation kit from Ambion. Next, the first strand cDNAs were synthesized using the Superscript First Strand cDNA synthesis kit from Applied Biosystems. For qPCR, specific primers were used. The primers used for human IL-6 mRNA were F 5′-AAA TTC GGT ACA TCC TCG ACG GCA-3′ and R 5′-AGT GCC TCT TTG CTG CTT TCA CAC-3′. The primers used for human GAPDH were F 5′-TCG ACA GTC AGC CGC ATC TTC TTT-3′ and R 5′-ACC AAA TCC GTT GAC TCC GAC CTT-3′. The quantified levels of mRNA or miRNA were presented as relative expression. In this analysis, untreated cancer cells were used as experimental controls, and the expression of RNU6B or GAPDH was used as an endogenous control, as previously described [82].

### 4.9. Human IL-6 Specific Sandwich ELISAs

The secretion levels of IL-6 protein in the culture supernatant of treated or non-treated cancer cells were quantified using human IL-6 specific sandwich ELISA kits in accordance with the manufacturers’ instructions (GenWay Biotech, San Diego, CA, USA).

### 4.10. Western Immunoblotting

The secretion levels of IL-6 protein in the culture supernatant of treated or non-treated cancer cells were detected by Western immunoblotting using anti-human IL-6 specific antibodies (Cell Signaling Technology, Danvers, MA, USA) as described previously [82].

### 4.11. Nuclear Factor-Kappa B p65/p50 DNA Binding Activity Assays

Transcription Factor Immunoassay Kits for the quantification of activated levels of NF-κB p65 and p50 in nucleus of treated or untreated cancer cells were used in accordance with the manufacturers’ instructions (catalog # ab133128, Abcam, Waltham, MA, USA). A well-known NF-κB inhibitor, Bay 11-7082 (Catalog # B5556, Sigma-Aldrich, St. Louis, MO, USA) was also used in cancer cells for further verification of NF-κB activity.

## 5. Conclusions

This is the first study that determined novel therapeutic targets of gold nanoparticles in cancer biology. The novel findings show for the first time that gold nanoparticles can inhibit interleukin-6 mRNA/protein secretion by upregulating miR-26a-5p and deactivating the RelA and NF-κBp50 transcription pathways in cancer cells. These findings shed light on the molecular mechanisms underlying the protective effects of gold nanoparticles against BC.

## Data Availability

All data and materials used in this study are available with corresponding author and will be provided on reasonable request.
